# Modern Operation for Cataract

**Published:** 1871-08

**Authors:** 


*Modern operation for Cataract.* By HASKET DERBY, M. D.

This is the title of a very able lecture delivered by the author at the Harvard Medical School. It is claimed that the "peripheric linear extraction" as advocated by Von Graefe, is the most successful, both to the surgeon and patient.

